# Protocatechualdehyde induced tumor suppressive autophagy through AMPK/ULK1 signaling pathway in gastric cancer

**DOI:** 10.3389/fonc.2025.1563006

**Published:** 2025-04-07

**Authors:** Mingming Ren, Fangqi Ma, Mengmeng Qin, Xiaoyu Sun, Yi Wang, Xiaohong Zhu, Yan Xu, Nida Cao, Ruohan Zhao, Yunchao Zhang, Jiangchuan Zhu, Yongfu Pan, Aiguang Zhao

**Affiliations:** ^1^ Department of Oncology, Longhua Hospital, Shanghai University of Traditional Chinese Medicine, Shanghai, China; ^2^ Cancer Institute of Traditional Chinese Medicine, Longhua Hospital, Shanghai University of Traditional Chinese Medicine, Shanghai, China; ^3^ Department of Traditional Chinese Medicine, The First Affiliated Hospital of University of Science and Technology of China (USTC), Division of Life Sciences and Medicine, University of Science and Technology of China, Hefei, Anhui, China; ^4^ Department of Traditional Chinese Medicine, The Second People’s Hospital of Lianyungang, Lianyungang, Jiangsu, China

**Keywords:** gastric cancer, protocatechualdehyde, autophagy, AMPK, ULK1

## Abstract

**Background:**

Gastric cancer (GC) is one of the primary causes of cancer-related fatalities, which requires novel treatment including traditional Chinese medicine (TCM) to prolong survival. Protocatechualdehyde (PCA), a monomer from Chinese herbs, exhibits an anti-cancer effect by inhibiting proliferation and migration, or inducing apoptosis in various types of tumors. However, the anti-cancer effect and underlying mechanism of PCA in gastric cancer are still unclear.

**Methods:**

The cell proliferation ability was detected by the cell counting kit-8 (CCK-8) and colony formation. The occurrence of autophagy was observed by TEM (Tansmission electron microscopy) and immunofluorescence. The expression of proteins involved in AMPK/mTOC1 signaling pathway was detected by western blotting. Apoptosis and cell cycle analysis were determined through flow cytometry. A xenograft mouse model was employed to validate the anticancer effect of PCA *in vivo*.

**Results:**

PCA was first identified as a specific inhibitor to gastric cancer cells that significantly inhibited the proliferation of human gastric cancer cells MKN45 and AGS in a dose- and time-dependent manner, but not that of human gastric epithelial cells. Furthermore, PCA induced tumor suppressive autophagy in both gastric cancer cells, and blockage of the autophagy by silencing ATG5 can partially reverse the proliferation inhibition of PCA. Mechanistically, PCA induced-autophagy was largely dependent on the activation of the AMPK/ULK1 signaling pathway, and blockage of the pathway through AMPK specific inhibitor Compound C (Com C) or siRNAs targeting ULK1 prevented the occurrence of autophagy and partially reversed the proliferation inhibition induced by PCA. In addition, PCA significantly suppressed the growth of gastric cancer in the gastric cancer xenograft mouse model by activating key proteins related to the AMPK/ULK1 signaling pathway of autophagy.

**Conclusion:**

These findings demonstrated that PCA inhibited gastric cancer by inducing tumor suppressive autophagy through the AMPK/ULK1 signaling pathway. PCA may serve as a novel candidate for the treatment of gastric cancer.

## 1 Introduction

Gastric cancer (GC) is one of the most common malignant tumors of the digestive system, ranking fifth in the incidence and mortality of malignant tumors worldwide (1). In clinic, most of the gastric cancer patients are diagnosed at advanced stage with poor prognosis due to the lack of improvement in early screening, as well as the high heterogeneity of gastric cancer (2, 3). Surgery, radiotherapy and chemotherapy are commonly used in the clinical treatment of gastric cancer. In addition, Traditional Chinese medicine (TCM) monomers such as paclitaxel and camptothecin are also one of the essential means of comprehensive treatment of gastric cancer (4). In recent years, TCM monomers have become a valuable source for the development of therapeutic agents for various complex diseases, including gastric cancer. Therefore, to screen and identify the monomer compounds with clear therapeutic effects and mechanisms against gastric cancer might serve as an essential direction in the field of TCM.

Protocatechualdehyde (PCA) is a polyphenolic widely derived from various medicinal plants, including Stenoloma chusana, Pinellia ternate and Salvia miltiorrhiza. Due to its multiple biological activities, PCA has been used in a series of diseases, including cancer. Previous studies have demonstrated that PCA inhibited the growth and migration of breast cancer cells (5). Furthermore, PCA induced cell cycle arrest and apoptosis in human colorectal cancer cells (6) and melanoma cells (7). However, the effect and underlying mechanisms of PCA in gastric cancer have not been explored.

Autophagy is an internal cleaning mechanism that can decompose and recycle old, damaged, or excess materials in mammalian cells (8). A series of stimuli including hypoxia, nutrient starvation, and low energy may induce autophagy through precise regulatory signaling pathways. Among them, the mammalian target of rapamycin (mTOR) and unc-51 like kinase 1 (ULK1) are the central regulators of autophagy in eukaryotes (9, 10). The mTOR is a threonine and serine kinase that can form two different complexes named mTORC1 and mTORC2 (11). Raptor (regulatory protein associated with mTOR), is one of the three core components of mTORC1 and is required for the correct subcellular localization of mTORC1 (12, 13). Under nutrient and growth factor-deprived conditions, the activity of mTORC1 is inhibited during the induction of autophagy through the regulation of the ULK1 complex (14). ULK1 complex is at least composed of ULK1, autophagy-related gene 13 (ATG13), and focal adhesion kinase family-interacting protein of 200 kDa (FIP200), which is important for maintaining the stability and kinase activity of ULK1 (15). ULK1 is required for autophagosome formation, which also can be activated by AMP-activated protein kinase (AMPK) by phosphorylation during nutrient and energy deficiency, thereby promoting autophagy (16).

Previous studies implicated that the role of autophagy during tumorigenesis and progression remains controversial. On one hand, autophagy promotes the survival and proliferation of tumor cells, and targeting autophagy is undoubtedly an excellent strategy for cancer treatment. On the other hand, autophagy may also play a role in tumor suppression by knocking out key genes of autophagy, such as BECN1 (17) and ATG7 (18). Therefore, exploring specific autophagy regulators suitable for *in vivo* use is the direction of future in-depth research.

In this study, we for the first time demonstrated PCA was a specific inhibitor to gastric cancer that triggered tumor suppressive autophagy through the AMPK/ULK1 signaling pathway. The study will provide theoretical support for identifying PCA as a potential treatment option for gastric cancer.

## 2 Materials and methods

### 2.1 Cell lines, culture and reagents

The human gastric cancer cells MKN45 and AGS, HEK293T, and human gastric epithelial cells GES-1 were obtained from the Cell Bank of the Chinese Academy of Sciences (Shanghai, China). MKN45 and GES-1 cells were cultured in RPMI-1640 medium (BasalMedia, Shanghai, China), HEK293T cells were cultured in DMEM medium (BasalMedia, Shanghai, China), and AGS cells were cultured in F12K medium (BasalMedia, Shanghai, China). The medium contained 10% fetal bovine serum (FBS, ExCell Bio, China) and 1% penicillin-streptomycin solution (Gibco, USA). Cells were maintained in a controlled environment at 37°C with 5% CO2. PCA, Chloroquine (CQ), Bafilomycin A1 (Baf-A1), 3-methyladenine(3-MA), Compound C (Com C), Ferrostatin-1 (Fer-1), and Necrosulfonamide (NSA) were purchased from MCE (MedChemExpress, Shanghai, China). Necrostatin-1 (Nec-1) and Z-VAD-FMK (Z-VAD) were purchased from Beyotime Biotechnology (Shanghai, China). Puromycin was obtained from Invitrogen and polybrene was from Santa Cruz.

### 2.2 Cell viability and clonogenic survival assay

Cells were seeded in 96-well plates (3×10^3^ cells/well) and treated with DMSO or PCA. Cell viability was measured with cell counting kit-8 (CCK-8, ShareBio, Shanghai, China) by a microplate reader (BIOTEK, USA). For the clonogenic assay, 500 cells were seeded in 12-well plates and then were treated with indicated concentration of PCA for 48 h, and cultured for 10 days. The colonies were fixed with 4% paraformaldehyde for 30 minutes and stained with 0.1% crystal violet. Colonies comprising 50 cells or more were observed under a gel imager (Bio-rad, USA).

### 2.3 Apoptosis assay and cell cycle assay

Cells were seeded in a 6-well plate and treated with the indicated concentration of PCA for 72 h. After treatment, apoptosis and cell cycle were determined using an Annexin V-fluorescein isothiocyanate/propidium iodide (Annexin V-FITC/PI) apoptosis kit and PI staining kit (Share Biosciences, Shanghai, China) by FACS analysis (Beckman Coulter, USA) according to the manufacturer’s instructions, respectively.

### 2.4 Western blotting

The total protein of cells and tissues was extracted and determined by the BCA protein assay kit (Epizyme, Shanghai, China). Equal amounts of proteins were loaded onto 10% and 12.5% SDS-PAGE gels and transferred to methanol-activated PVDF membranes (Millipore, Germany). Membranes were then blocked with 5% skim milk in Tris-Buffered Saline with 0.1% Tween (TBST) at room temperature for 2 h. Subsequently, membranes were incubated with primary antibodies and conjugated with secondary antibodies. Finally, signals were obtained by Tanon 5200 visualizer (Tanon, Shanghai, China). The primary antibodies AMPK, p-AMPKa (Thr172), ULK1, p-ULK1 (Ser555), S6K, p-S6K (Thr389), mTOR, p-mTOR (Ser2448), p62, ATG5, Beclin1, RAPTOR, p-RAPTOR (Ser792) were purchased from Cell Signaling Technology. PKA, TSC2, and Rheb antibodies were obtained from Santa Cruz Biotechnology. LKB1 antibody was from Selleck. The LC3B antibody was from Proteintech. The β-actin antibody was from Beyotime.

### 2.5 RNA interfering

Cells were transfected with short interfering RNA oligonucleotides by Lipofectamine RNAiMAX (Invitrogen, USA) following the manufacturer’s protocol. All siRNAs were synthesized by GenePharma (Shanghai, China), and sequences were summarized as follows:

si-ULK1-1, 5′-CGCCUGUUCUACGAGAAGA-3′;

si-ULK1-2, 5′-GGCUGAAUGAGCUGUACAA-3′;

si-ATG5-1,5′-GGUUUGGACGAAUUCCAACUUGUUU-3′;

si-ATG5-2, 5′-GAUCACAAGCAACUCUGGAUGGGAU-3′;

si-Control, 5′-UUCUCCGAACGUGUCACGU-3′.

### 2.6 Transmission electron microscopy

MKN45 and AGS cells were treated with DMSO or indicated concentration of PCA, then fixed with 2.5% glutaraldehyde and 1% osmium tetroxide, dehydrated with ethanol, embedded in epoxy resin, and cut into 70nm ultra-thin sections with an ultramicrotome (Leica EM UC7, Germany). The sections were treated with uranium–lead double staining, mounted on transmission electron microscopy grids, and examined using a transmission electron microscope for high-resolution imaging of cellular ultrastructure (Joel JEM-1230, Japan).

### 2.7 Establishment of EGFP-LC3B stable cell lines

Human *LC3B* gene was cloned to EGFP-C3 plasmid, then inserted to pCDH-CMV-MCS-EF1-Puro plasmid and co-transfected with packaging plasmid psPAX2 and envelop plasmid PM2D.G in HEK293T cells by Lipofectamine 2000 (Invitrogen, USA). After 48h transfection, the lentivirus supernatant was collected to infect MKN45 and AGS cells with 10 μg/mL polybrene (Santa Cruz, USA). The EGFP-LC3B positive cells were selected by 4.0 μg/mL puromycin (Invitrogen, USA) for 2 weeks, then treated with DMSO or PCA for 72 h, and finally imaged with fluorescence microscopy (KEYENCE BZ-X800L, Japan).

### 2.8 Tumor xenograft model

A subcutaneous tumor model of gastric cancer was established using MKN45 cells. Male 4-6-week-old athymic balb/c nude mice provided by the Experimental Animal Center of Shanghai University of Traditional Chinese Medicine were housed in a specific pathogen-free environment. MKN45 cells(2*10^6^) were suspended in 100 μL PBS, and then subcutaneously injected into the right axillae of each mouse. After 4 days, mice were randomly divided into 3 groups: control group, PCA low group (20 mg/kg), and PCA high group (40 mg/kg), and were intraperitoneally injected each other day for 21 consecutive days. The tumor volume and body weight were measured every 4 days. Tumor volume was calculated using the formula: tumor volume = (length * width^2)/2, where length is the longest dimension and width is the dimension perpendicular to the length. At the end of the experiment, mice were anesthetized with pentobarbital sodium and then euthanized by cervical dislocation. Tumor tissues were harvested, photographed, weighed fixed in 4% paraformaldehyde, and stained with Hematoxylin-Eosin (H&E) staining. The pathological change of liver and kidney tissues of mice was assessed by H&E staining. All procedures of animal experiments were conducted in compliance with standard ethical guidelines and approval of the Experimental Animal Ethics Committee of Shanghai University of Traditional Chinese Medicine (approval number: PZSHUTCM2312040004).

### 2.9 Immunohistochemistry staining

Tumor tissues were deparaffinized, rehydrated, and washed in 1% Tween PBS, then treated with 3% hydrogen peroxide and blocked with 10% goat serum for 1 h at 37°C. Tumor tissues were incubated with Ki-67 primary antibody (Abcam, USA) and secondary antibody. Subsequently, the sections underwent DAB staining, hematoxylin counterstaining, alcohol dehydration, xylene clearing, and coverslipping. Finally, the images were captured using a microscope (Nikon DS-Fi2, Japan).

### 2.10 Statistical analysis

The data were presented as the mean ± standard deviation (SD). Student’s t-test was used for comparison between the two groups. A one-way ANOVA was used for comparison between multiple groups. All analyses were performed using GraphPad Prism version 8.0 (GraphPad Software, California, USA). Four levels of significance were used for all tests,∗p < 0.05, ∗∗p < 0.01, and ∗∗∗p < 0.001, n.s. denotes no significance.

## 3 Results

### 3.1 PCA was a specific inhibitor to gastric cancer cells

**Figure 1:**
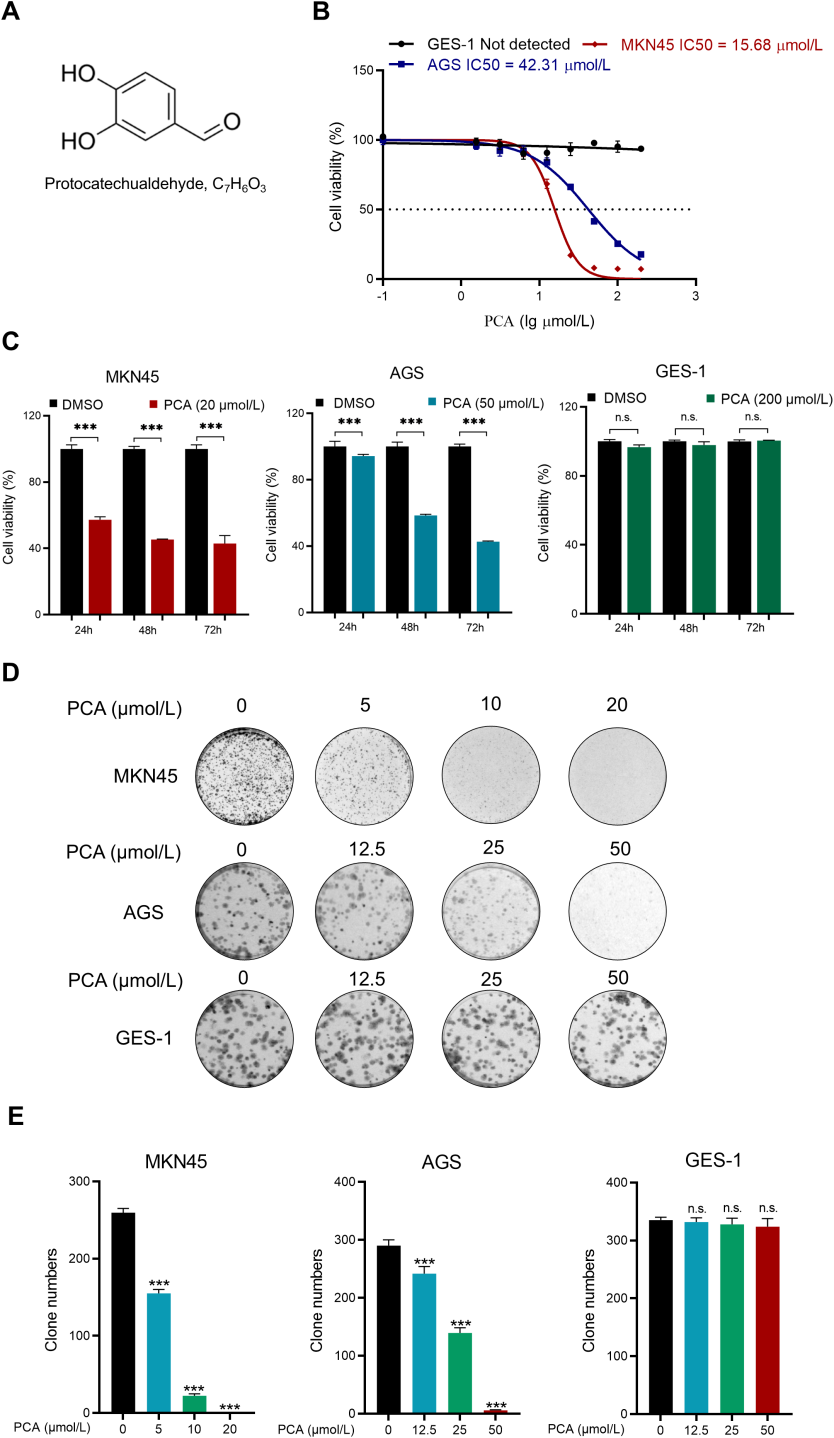
PCA was a specific inhibitor to human gastric cancer cells, but not normal mucosal cells. **(A)**The chemical structure of PCA. **(B)** Human gastric cancer cells MKN45, AGS and human gastric mucosal cell GES-1 were treated with PCA at 0,1.56, 3.12, 6.25, 12.5, 25, 50, 100, 200 (μmol/L) for 72 h, and the IC50 was determined by CCK-8 assay. **(C)** MKN45, AGS and GES-1 cells were treated with PCA at indicated concentrations for 24 h, 48 h and 72 h, and cell viability was detected by CCK-8 assay. **(D, E)** Representative images and statistical data were shown for the inhibition of colony formation by PCA. ^∗∗∗^p < 0.001, n.s. denotes no significance.

To assess the anti-cancer effect of PCA, CCK-8 assays were employed to study the cell viability of gastric cancer cells. The chemical structure of PCA is shown in **Figure 1A**. As shown in **Figures 1B, C**, PCA significantly inhibited the proliferation of human gastric cancer cells MKN45 and AGS in a dose-dependent manner, but not that of human gastric epithelial cells GES-1. The half-maximal inhibiting concentration (IC50) values of PCA in AGS and MKN45 were 15.68 μmol/L and 42.31 μmol/L, respectively. Furthermore, PCA significantly inhibited the proliferation of gastric cancer cells in a time-dependent manner, but not that of GES-1 cells under the maximal dose (200 μmol/L) at indicated times. These results indicated that PCA specifically inhibited the proliferation of gastric cancer cells, but not normal gastric epithelial cells, the phenomenon was further confirmed by the colony formation assay (**Figures 1D, E**). The above data indicated that PCA was a specific inhibitor to gastric cancer cells.

### 3.2 PCA neither induced various forms of cell death nor affected cell cycle in gastric cancer cells

**Figure 2:**
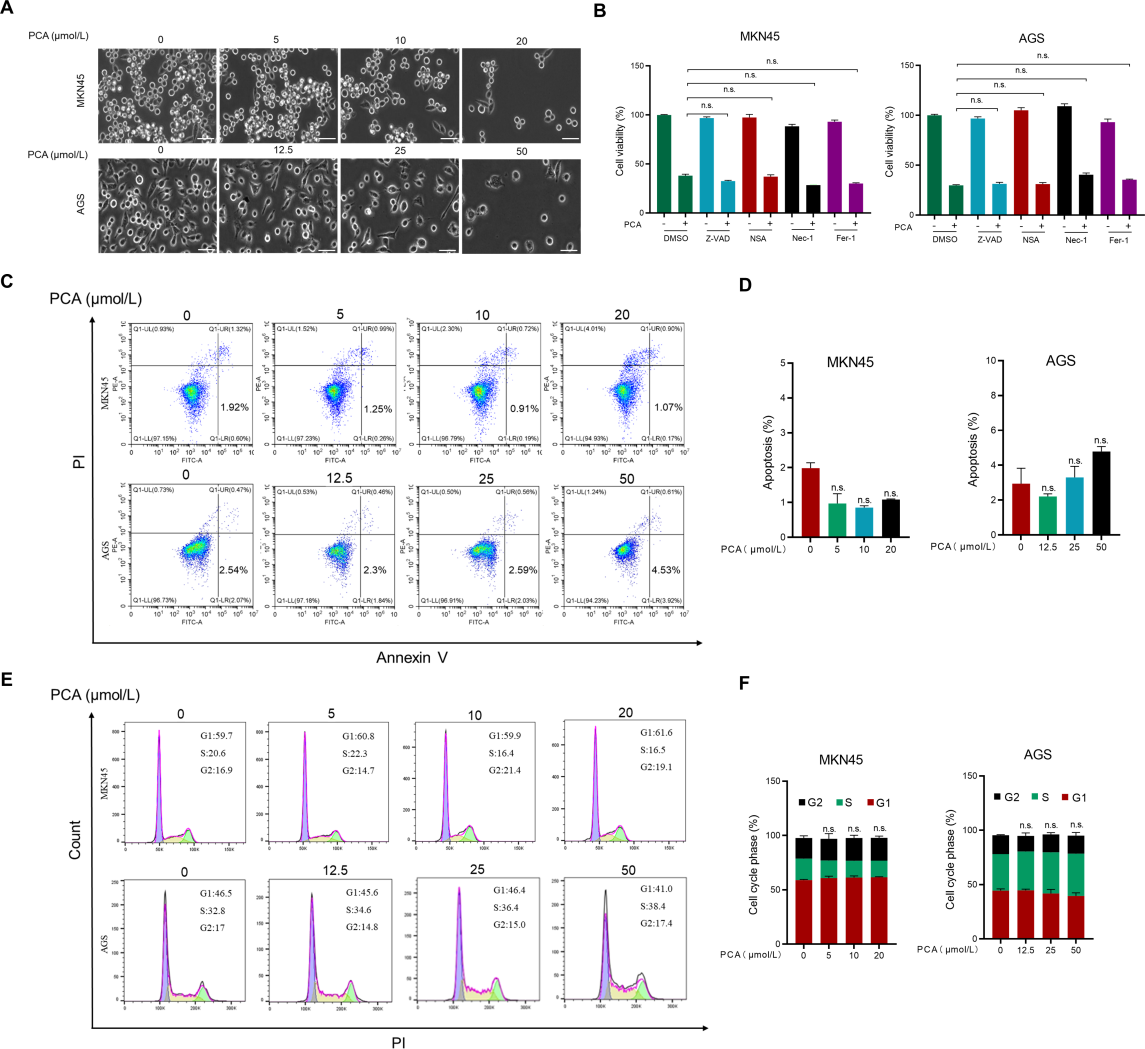
PCA neither induced various forms of cell death nor affected cell cycle in gastric cancer cells. **(A)** Representative images of the morphology change of MKN45 and AGS cells treated with PCA at indicated concentrations for 72 h (scale bar = 50 μm). **(B)** MKN45 and AGS cells were incubated with Z-VAD (20 μmol/L), Nec-1 (50 μmol/L), NSA (2 μmol/L), and Fer-1 (1 μmol/L) or combined with PCA at 20 μM and 50 μM for 72 h, respectively, and cell viability was determined by CCK-8 assay. **(C, D)** MKN45 and AGS cells were incubated with PCA at indicated times for 72 h, then stained with an Annexin-V-FITC/PI apoptosis detection kit and analyzed with FCAS. **(E, F)** MKN45 and AGS cells were incubated with PCA for 72 h, followed by PI staining, finally detected by FACS. n.s. denotes no significance.

Under the microscope, the morphology of gastric cancer cells did not significantly change in response to PCA (**Figure 2A**). To further investigate the underlying mechanism of PCA on the proliferation inhibition in gastric cancer cells, inhibitors of various types of cell death were primarily employed in the following studies. As shown in **Figure 2B**, none of the inhibitors were able to reverse the proliferation inhibition of PCA in both gastric cancer cells, indicating that PCA did not induce apoptosis, ferroptosis or necroptosis. Furthermore, results of Annexin V/PI staining (**Figures 2C, D**) confirmed that PCA did not initiate apoptosis during the treatment. In addition, PCA did not affect cell cycle in both gastric cancer cells through flow cytometry analysis (**Figures 2E, F**).

### 3.3 PCA induced autophagy in gastric cancer cells

**Figure 3:**
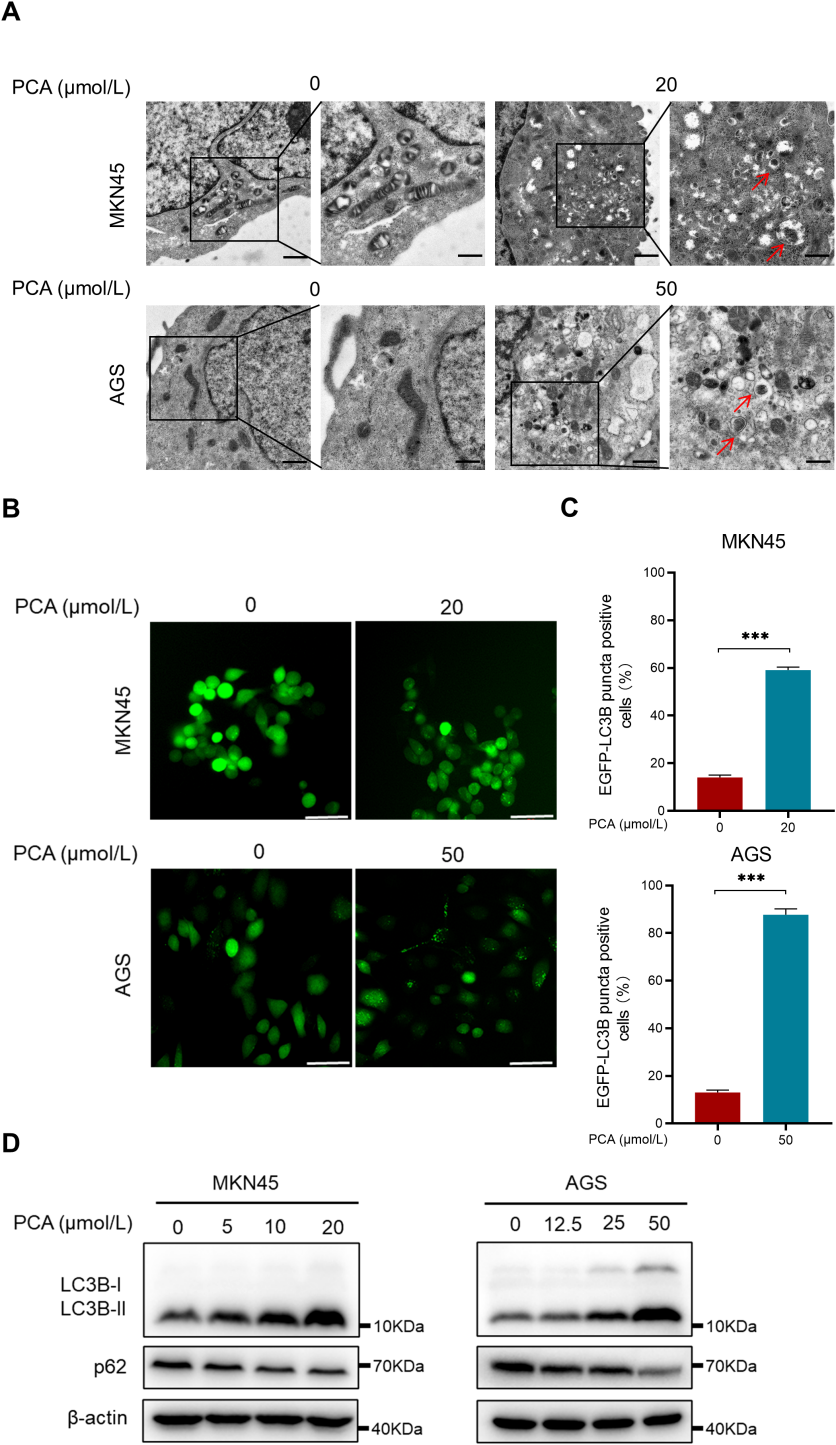
PCA induced autophagy in gastric cancer cells. **(A)** MKN45 and AGS cells were treated with indicated concentration of PCA, then imaged by transmission electron microscopy (scale bar = 1 μm or 500 nm), red arrows indicate autolysosomes. **(B, C)** EGFP-LC3B stable cells MKN45 and AGS were treated with indicated concentration of PCA, then imaged by fluorescence microscope. The EGFP-LC3B puncta positive cells were quantified (scale bar = 50 μm). **(D)** MKN45 and AGS cells were treated with PCA at indicated concentration of PCA for 72 h, proteins were extracted and detected by western blotting. ^∗∗∗^p < 0.001, n.s. denotes no significance.

Interestingly, the ability of gastric cancer cells to engulf intracellular solutes or organelles in the lysosome significantly enhanced in response to PCA by using transmission electron microscopy (**Figure 3A**). Furthermore, PCA triggered remarkable accumulation of EGFP-LC3B punta in both gastric cancer cells under fluorescence microscope (**Figures 3B, C**), suggesting the occurrence of autophagy. In addition, PCA induced the formation of autophagy marker LC3B-II from LC3B-I and the down-regulation of p62 (**Figure 3D**), which further supported that PCA inducted autophagy during the experiment. Taken together, these data supported that PCA induced autophagy in gastric cancer cells.

### 3.4 PCA induced-autophagy played a tumor suppressive role in gastric cancer cells

**Figure 4:**
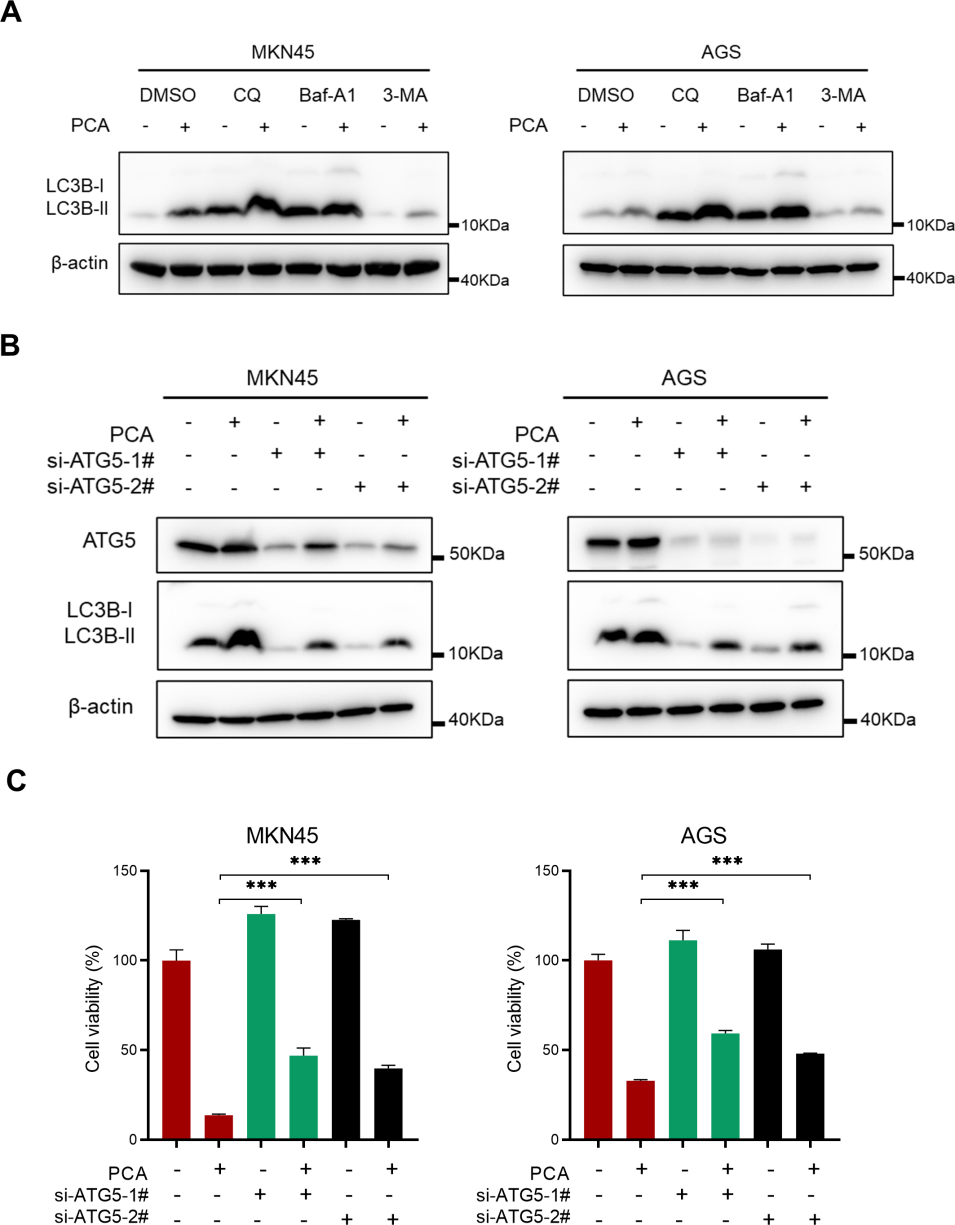
Blockage of autophagy partially rescued the proliferation inhibition by PCA. **(A)** MKN45 and AGS cells were treated with PCA at 10 μmol/L and 20 μmol/L for 12 h, respectively, then treated with autophagy inhibitors CQ (20 μmol/L), Baf-A1 (40 nmol/L) and 3-MA (10 μmol/L) for another 12 h, proteins were extracted and detected by western blotting. **(B, C)** MKN45 and AGS cells were transfected with control or two siRNA sequences of ATG5 and then treated with PCA at indicated concentrations for 72 h, proteins were extracted and detected by western blotting, and cell viability was determined by CCK-8 assay. ^∗∗∗^p < 0.001, n.s. denotes no significance.

To further examine the autophagy flux by PCA, classical autophagy inhibitors including Baf-A1, CQ, and 3-MA were employed in the study. As shown in **Figure 4A**, the combination treatment resulted in a greater accumulation of LC3B-II compared to either PCA or the autophagy inhibitors alone, suggesting the autophagy flux remained intact and PCA induced an autophagic response. To validate the role of autophagy during PCA-inducing proliferation inhibition, the essential protein of autophagy ATG5 was silenced. As shown in **Figure 4B**, siRNAs effectively silenced ATG5 and significantly inhibited the formation of LC3B-II, suggesting the autophagy process was blocked. Moreover, blockage of autophagy significantly reversed the proliferation inhibition by PCA (**Figure 4C**). These results indicated that PCA induced autophagy is lethal to gastric cancer cells.

### 3.5 PCA induced autophagy through AMPK/ULK1 signaling pathway

**Figure 5:**
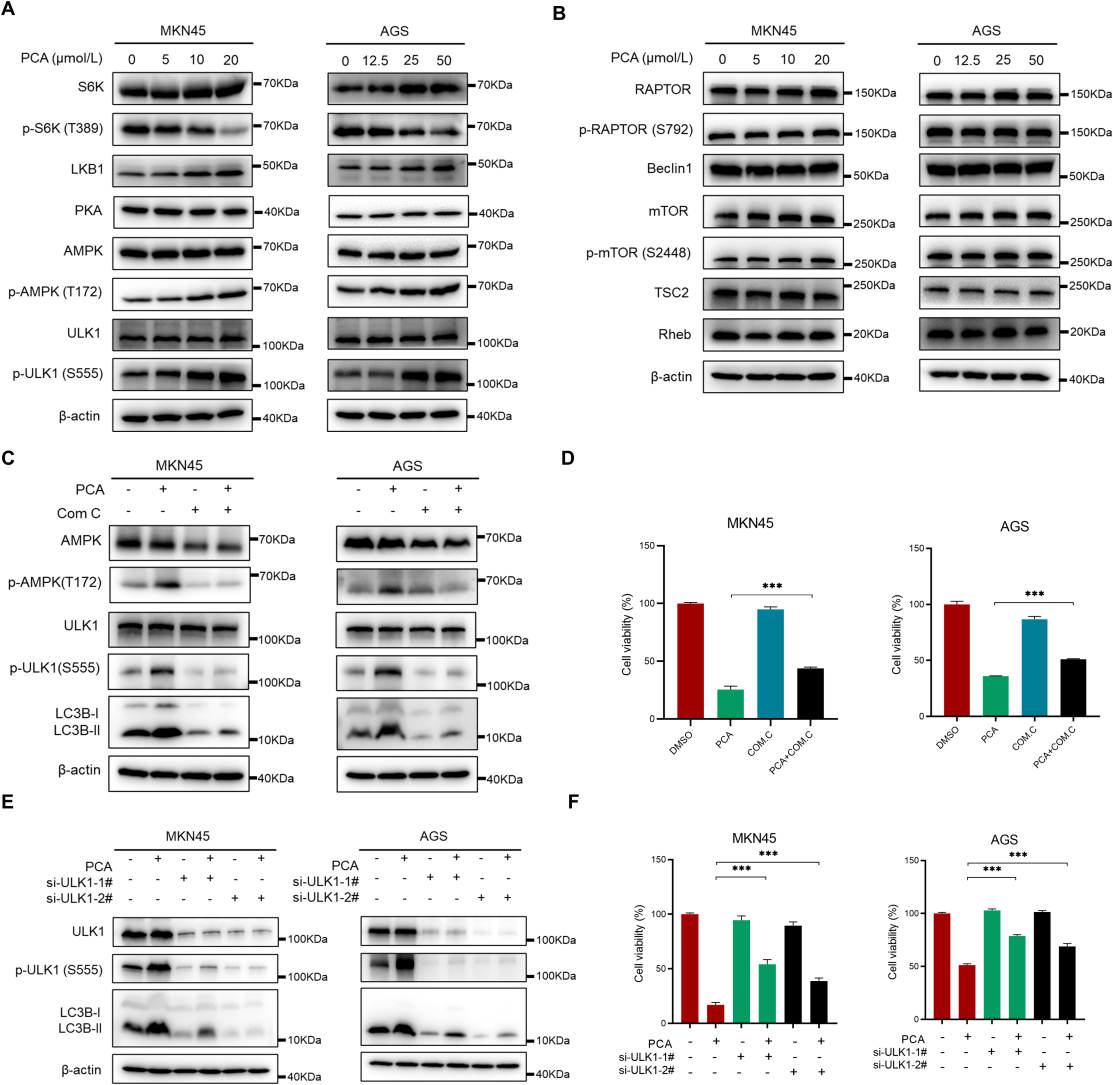
PCA induced autophagy through AMPK-ULK1 pathway in gastric cancer cells. **(A, B)** MKN45 and AGS cells were treated with PCA at indicated concentration of PCA for 72 h, proteins were extracted and detected by western blotting. **(C, D)** MKN45 and AGS cells were treated with 20 μmol/L and 50 μmol/L PCA, and 1 μmol/L and 0.2 μmol/L AMPK inhibitor Com C alone or in combination for 72 h, respectively. Proteins were extracted and detected by western blotting and cell viability was determined by CCK-8 assay. **(E, F)** MKN45 and AGS cells were transfected with control or two siRNA sequences of ULK1 and treated with PCA at indicated concentrations for 72 h, proteins were extracted and detected by western blotting, and cell viability was determined by CCK-8 assay. ^∗∗∗^p < 0.001, n.s. denotes no significance.

To investigate the mechanism of autophagy induction by PCA, the essential proteins of the AMPK/ULK1/mTOR signaling pathway were primarily detected by western blotting. As shown in **Figures 5A, B**, PCA significantly induced the up-regulation of phosphorylation of AMPK and ULK1, and the down-regulation of phosphorylation of S6K the classical downstream effector of autophagy, supporting that AMPK/ULK1 signaling pathway was activated during PCA-induced autophagy. However, the protein level of Beclin1, p-mTOR, and p-RAPTOR did not change during the experiment, suggesting that the mTORC1 complex was not involved in the process. Furthermore, the application of AMPK specific inhibitor Com C significantly inhibited the activation of AMPK and ULK1, as well as the formation of LC3B-II, which meanwhile partially reversed the proliferation inhibition of PCA (**Figures 5C, D**), indicating PCA induced autophagy was largely depended on AMPK activation. In addition, knockdown of ULK1 by two validated siRNAs significantly rescued the proliferation inhibition of PCA (**Figures 5E, F**), supporting that PCA induced ULK1-dependent autophagy. Collectively, these results indicated that PCA induced-autophagy relied on the AMPK/ULK1 signaling pathway.

### 3.6 PCA suppressed gastric cancer *in vivo* by inducing autophagy

**Figure 6:**
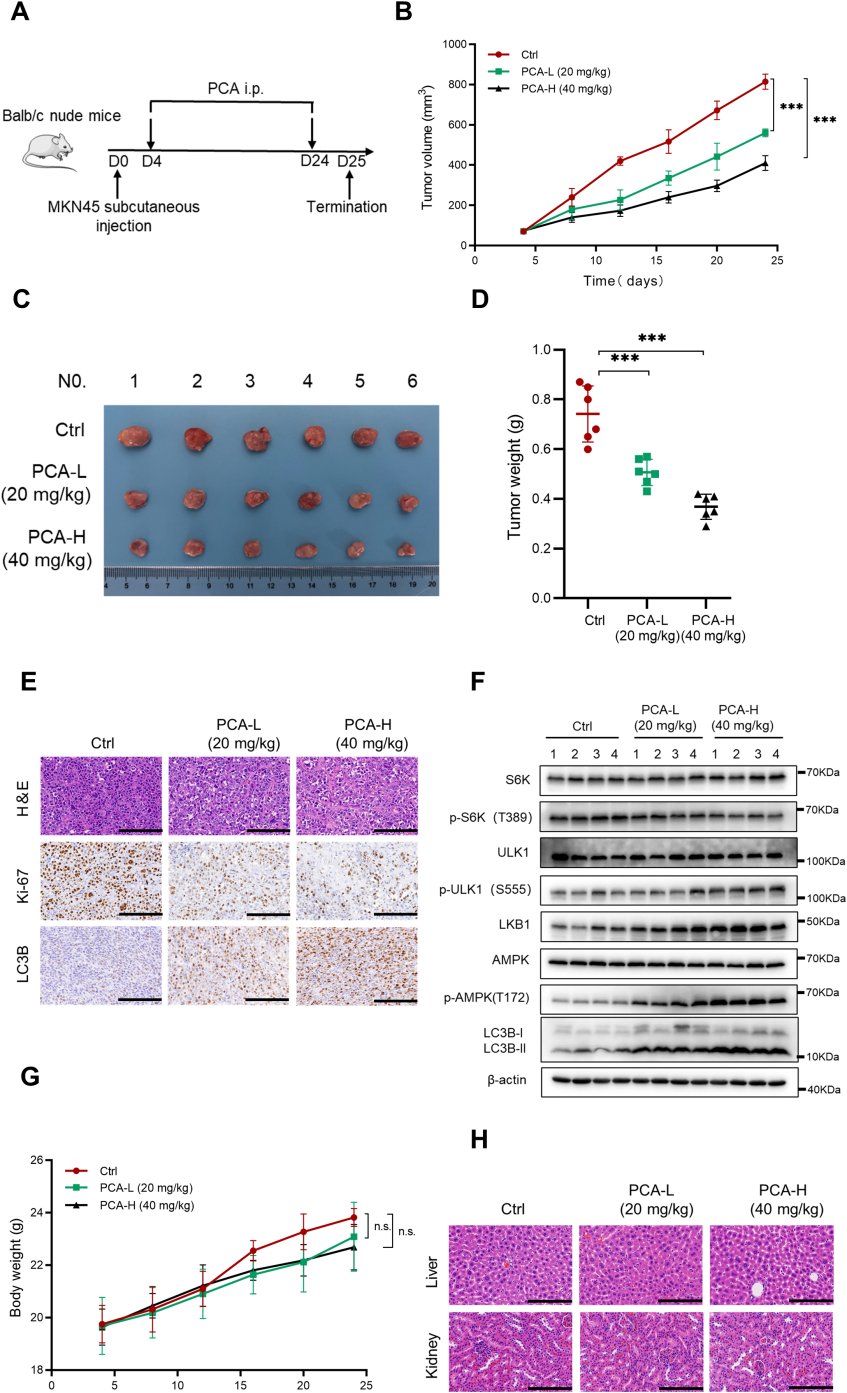
PCA suppressed gastric cancer in vivo by inducing autophagy. **(A)** Nude mice were subcutaneously transplanted with MKN45 cells and treated with PCA as indicated in materials and methods (n = 6). **(B)** Tumor size was detected with a caliper, and the volume was calculated to construct a growth curve. **(C)** Images of tumors from nude mice after the treatment (n = 6). **(D)** Tumor weight was recorded when experiments were terminated. **(E)** Representative images of H&E staining and IHC staining of Ki-67 and LC3B in tumor tissues (scale bar = 100 μm). **(F)** The expression of proteins in different groups were evaluated using western blotting (n = 4). **(G)** The body weight of nude mice was recorded every four days during the entire experiment (n = 6). **(H)** Representative images of H&E staining in liver and kidney of mice (scale bar = 100 μm). ^∗∗∗^p < 0.001, n.s. denotes no significance.

Based on the *in vitro* results, we evaluated the anti-cancer effect of PCA by establishing a xenograft tumor model with MKN45 cells *in vivo* (**Figure 6A**). Compared to the control group, the tumor volume and weight were significantly suppressed by the intraperitoneal injection with high or low dosage of PCA (**Figures 6B–D**). H&E and Ki-67 staining revealed that PCA significantly suppressed the growth of gastric cancer cells, meanwhile, LC3B staining indicated PCA induced autophagy during the experiment (**Figure 6E**). Furthermore, western blotting analysis of tumor tissues showed that PCA induced the up-regulation of p-ULK1, p-AMPK and formation of LC3B-II, as well as the down-regulation of p-S6K (**Figure 6F**), which was consistent with *in vitro* results. These results strongly supported that PCA suppressed gastric cancer by inducing AMPK/ULK1-dependent autophagy. In addition, the administration of PCA neither significantly affected the body weight of mice (**Figure 6G**), nor caused pathological alterations of the liver and kidney of mice (**Figure 6H**), suggesting that PCA exhibited no obvious toxicity during the treatment. Taken together, these data indicated that PCA was a molecule with high safety that suppressed gastric cancer by inducing autophagy.

## 4 Discussion

Gastric cancer is one of the major malignant tumors that pose a significant threat to human health, resulting in substantial economic and social burdens. An increasing body of clinical data demonstrates that TCM plays an indispensable role in gastric cancer treatment due to its unique advantages, including multi-target engagement, regulation of multiple signaling pathways, and minimal toxic side effects (19). PCA, a monomer derived from the Chinese herbal with various biological activities (20–25), exerts anti-cancer effects through regulating proliferation, migration, cell cycle, and apoptosis (5, 6). In the study, we for the first time found that PCA can selectively inhibit the proliferation of gastric cancer cells, and its underlying main mechanism was to induce tumor suppressive autophagy.

Autophagy is a process by which cells engulf cytoplasmic proteins or organelles for degradation in lysosomes to maintain intracellular homeostasis. Autophagy promotes tumor development by providing nutrients to cells (26), metabolic reprogramming (27), and immune escape (28, 29), thus blocking autophagy such as HCQ may serve as an anti-cancer strategy (30). Meanwhile, autophagy may also suppress tumorigenesis by maintaining genomic integrity, inhibiting inflammation and oxidative stress response (31, 32). In the study, we demonstrated that PCA induced tumor suppressive autophagy, and blocking autophagy by si-*ATG5* promotes survival in gastric cancer (**Figure 4**). Considering that autophagy may trigger various types of cell death, including apoptosis, necroptosis, and ferroptosis (33–36), inhibitors of these death pathways did not prevent the proliferation inhibition by PCA (**Figure 2B**), indicating that PCA did not induce these types of cell death in gastric cancer cells. Given that autophagy may initiate other modes of cell death, more experiments should be designed and conducted in the future.

In mechanism, PCA-induced autophagy was largely dependent on the AMPK/ULK1 signaling pathway, and blocking the pathway by AMPK specific inhibitor Com C or ULK1 silencing partially reversed the proliferation inhibition of PCA in gastric cancer. It is well-recognized that ULK1 was phosphorylated at Ser555 by AMPK under glucose or amino acid starvation-induced autophagy (37). Unexpectedly, the protein level of p-mTOR (Ser2446), p-Raptor (Ser792), and RheB did not change, suggesting mTORC1 and canonical autophagy pathway were not activated during the experiment. Moreover, the up-regulation of LKB1, the kinase responsible for AMPK activation (16) was observed in the experiment, suggesting PCA may trigger energy stress in the process. Thus, more effort should focus on the upstream of AMPK activation.

## 5 Conclusion

In summary, we conducted a comprehensive evaluation of the anti-cancer effect of PCA and explored its underlying mechanism in gastric cancer. Our findings for the first time revealed that PCA inhibited gastric cancer by inducing tumor suppressive autophagy through the AMPK/ULK1 signaling pathway. Given that PCA is a substance with various biological activities, identifying direct target proteins will further elucidate its anti-cancer mechanism.

## Data Availability

The original contributions presented in the study are included in the article/**Supplementary Material**. Further inquiries can be directed to the corresponding authors.

## Supplementary material

The Supplementary Material for this article can be foundonline at: https://www.frontiersin.org/articles/10.3389/fonc.2025.1563006/full#supplementary-material
