# Supplementary figures and images for: Protocatechualdehyde induced tumor suppressive autophagy through AMPK/ULK1 signaling pathway in gastric cancer
Source: Front Oncol. 2025 Apr 7;15:1563006. doi: 10.3389/fonc.2025.1563006 (PMC12009724; doi:10.3389/fonc.2025.1563006)

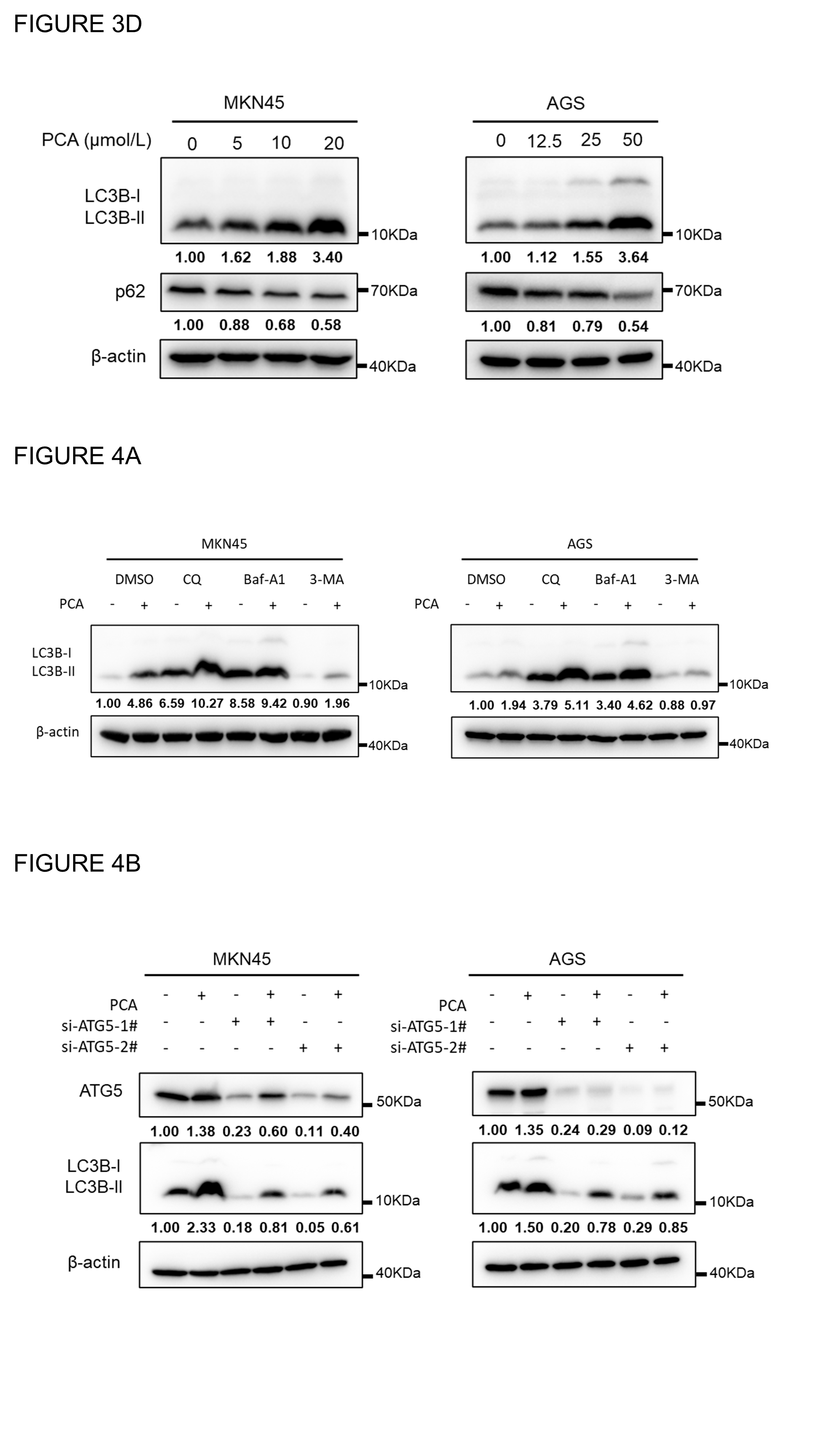

Supplement: Supplementary file 1 [file DataSheet1.zip › Revised_Supplementary file_1563006/Supplementary figure 1.TIF]

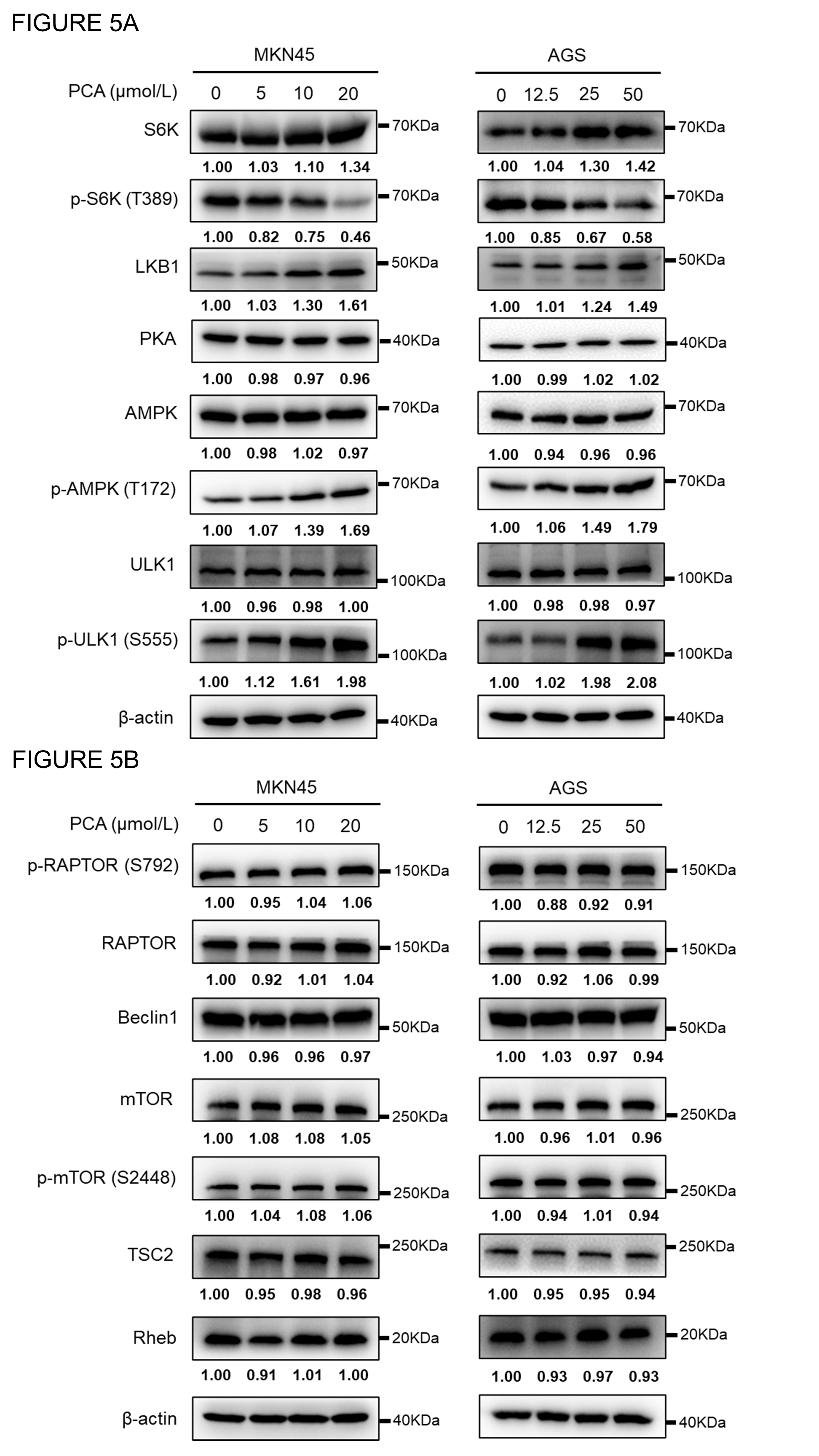

Supplement: Supplementary file 1 [file DataSheet1.zip › Revised_Supplementary file_1563006/Supplementary figure 2.TIF]

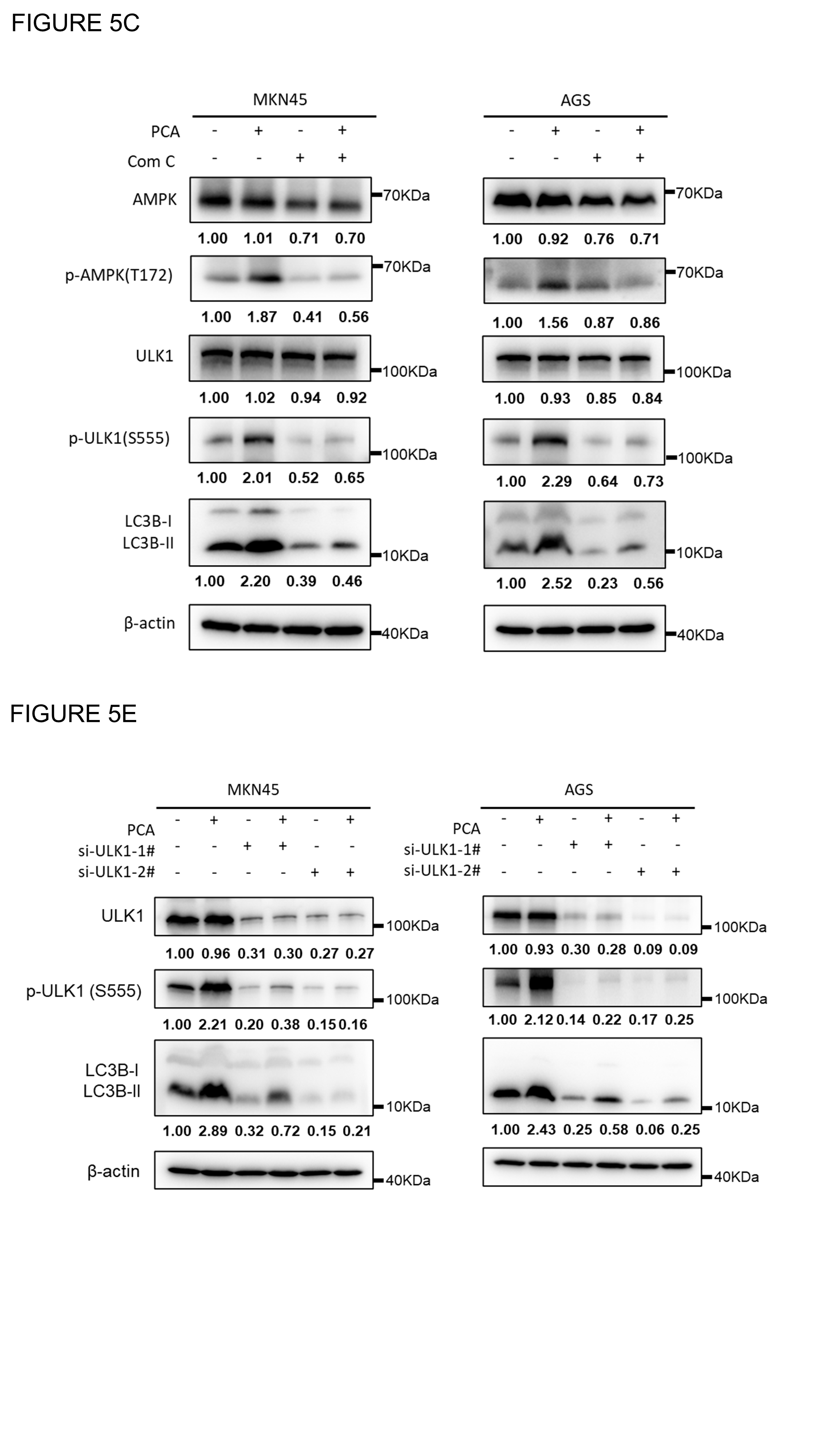

Supplement: Supplementary file 1 [file DataSheet1.zip › Revised_Supplementary file_1563006/Supplementary figure 3.TIF]

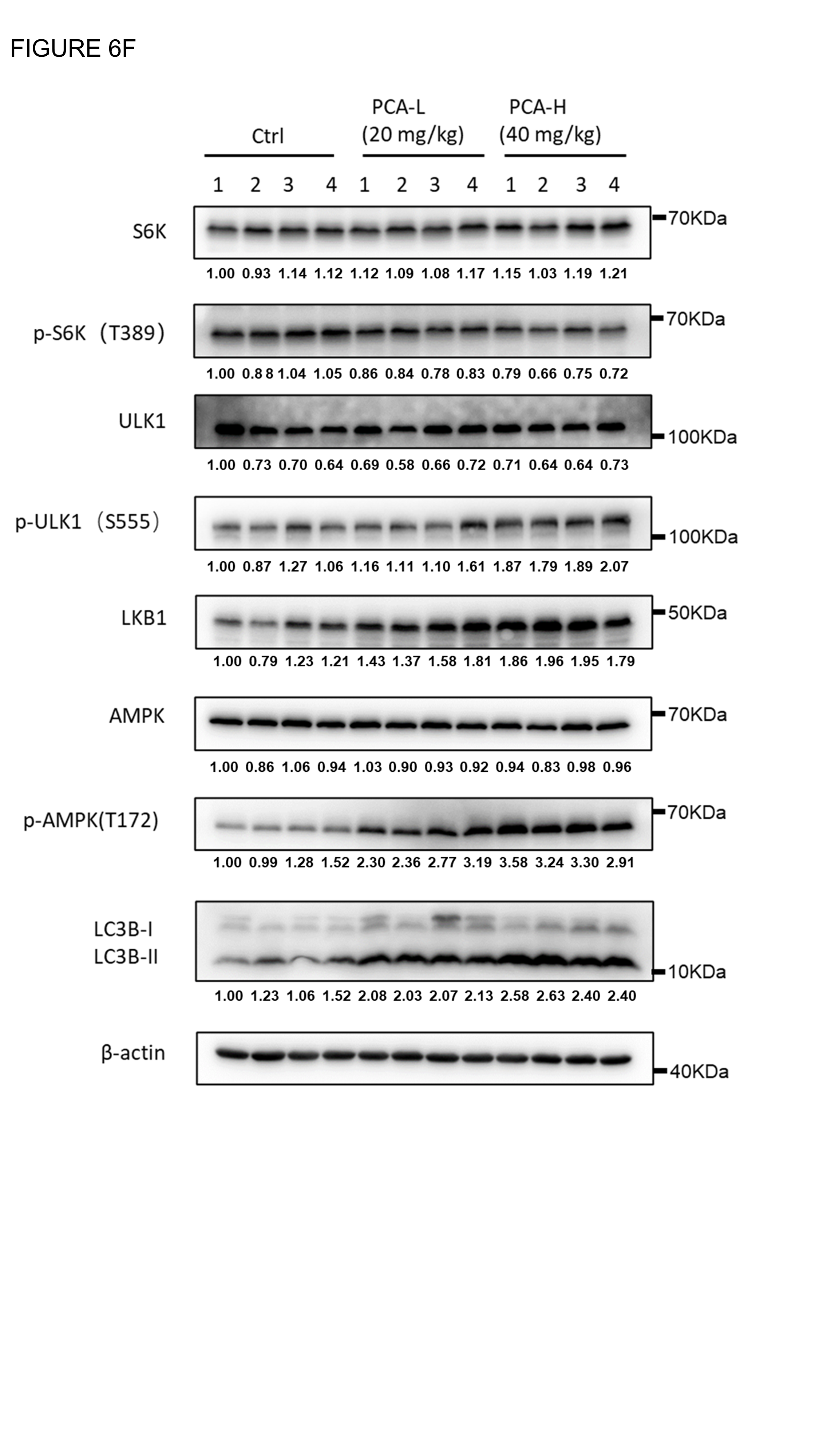

Supplement: Supplementary file 1 [file DataSheet1.zip › Revised_Supplementary file_1563006/Supplementary figure 4.TIF]
